# Quorum Sensing-Independent Cellulase-Sensitive Pellicle Formation Is Critical for Colonization of *Burkholderia glumae* in Rice Plants

**DOI:** 10.3389/fmicb.2019.03090

**Published:** 2020-01-17

**Authors:** Gi-Young Kwak, Okhee Choi, Eunhye Goo, Yongsung Kang, Jinwoo Kim, Ingyu Hwang

**Affiliations:** ^1^Department of Agricultural Biotechnology, Seoul National University, Seoul, South Korea; ^2^Division of Applied Life Science, Institute of Agriculture and Life Sciences, Gyeongsang National University, Jinju, South Korea; ^3^Research Institute of Agriculture and Life Sciences, Seoul National University, Seoul, South Korea

**Keywords:** *Burkholderia glumae*, pellicle, virulence, colonization, panicle blight, cellulose biosynthesis, quorum sensing

## Abstract

Bacteria form biofilms as a means to adapt to environmental changes for survival. Pellicle is a floating biofilm formed at the air–liquid interface in static culture conditions; however, its functional roles have received relatively little attention compared to solid surface-associated biofilms in gram-negative bacteria. Here we show that the rice pathogen *Burkholderia glumae* BGR1 forms cellulase-sensitive pellicles in a bis-(3′-5′)-cyclic dimeric guanosine monophosphate (c-di-GMP)- and flagellum-dependent, but quorum sensing (QS)-independent, manner. Pellicle formation was more favorable at 28°C than at the optimum growth temperature (37°C), and was facilitated by constitutive expression of *pelI*, a diguanylate cyclase gene from *B. glumae*, or *pleD*, the GGDEF response regulator from *Agrobacterium tumefaciens*. Constitutive expression of *pelI* or *pleD* raised the levels of c-di-GMP, facilitated pellicle formation, and suppressed swarming motility in *B. glumae*. QS-defective mutants of *B. glumae* formed pellicles, while flagellum-defective mutants did not. Pellicles of *B. glumae* were sensitive to cellulase but not to proteinase K or DNase I. A gene cluster containing seven genes involved in bacterial cellulose biosynthesis, *bcsD*, *bcsR*, *bcsQ*, *bcsA*, *bcsB*, *bcsZ*, and *bcsC*, homologous to known genes involved in cellulose biosynthesis in other bacteria, was identified in *B. glumae*. Mutations in each gene abolished pellicle formation. These results revealed a positive correlation between cellulase-sensitive pellicles and putative cellulose biosynthetic genes. Pellicle-defective mutants did not colonize as successfully as the wild-type strain BGR1 in rice plants, which resulted in a significant reduction in virulence. Our findings show that cellulase-sensitive pellicles produced in a QS-independent manner play important roles in the interactions between rice plants and *B. glumae*.

## Introduction

Bacterial biofilms are complex multicellular complexes embedded with self-producing extracellular materials such as polysaccharides, proteins, and nucleic acids (Donlan, 2002; Flemming et al., 2007). Bacterial biofilms are generally developed on diverse solid surfaces, and biofilm formed at the air–liquid interface is called floating biofilm or pellicle (Armitano et al., 2014). Under unfavorable growth conditions or in certain ecological niches, bacterial cells form complex biofilm structures for their survival (Williams and Cannon, 1989; Kovács and Dragoš, 2019). Aerotactic bacterial cells are coagulated at the air–liquid interface by flagellum-mediated motility, then switch their lifestyles to become sessile cells through bis-(3′-5′)-cyclic dimeric guanosine monophosphate (c-di-GMP)-mediated signal transduction systems (Römling et al., 2005, 2013; Römling and Amikam, 2006). In addition, bacterial quorum sensing (QS) often plays critical roles in biofilm formation (Hentzer et al., 2004; De Kievit, 2009; Guttenplan and Kearns, 2013). Formation of pellicles provides fitness and survival advantages (Boles and Singh, 2008); however, the roles of cellulosic pellicles in the natural environment are not well understood, especially in interactions between plant pathogenic bacteria and their hosts.

Common features of pellicles in gram-negative bacteria include that oxygen is critical to triggering pellicle formation, flagellar motility is important, and cellulose is a main component of the pellicle matrix (Spiers et al., 2003; Ude et al., 2006; Barken et al., 2008; Hölscher et al., 2015). One variety of Proteobacteria inhabiting diverse ecological niches comprises bacterial cellulose producers (O’Toole et al., 2000; Sutherland, 2001). Bacterial cellulose facilitates intimate interactions between bacterial cellulose producers and various components present in the environment (Yamamoto et al., 2011). Particularly for plant- or animal-associated bacteria, cellulosic biofilms are important for close interactions with their hosts (Pérez-Mendoza et al., 2014; Augimeri et al., 2015; Yang et al., 2019). Formation of bacterial cellulosic pellicle is accomplished by five main proteins encoded by bacterial cellulose biosynthetic (*bcs*) genes, *bcs A*, *B*, *C*, *D*, and *Z* (Saxena et al., 1994; Le Quéré and Ghigo, 2009). In addition to cellulose biosynthetic genes, c-di-GMP plays a key role in the regulation of bacterial biofilm formation (Hengge, 2009; Houry et al., 2010; Römling et al., 2013). c-di-GMP is biosynthesized by diguanylate cyclases (DGCs) that often possess a GGDEF motif, and then hydrolyzed into 5′-phosphoguanylyl-(3′-5′)-guanosine by phosphodiesterases (PDEs) carrying an EAL domain and degraded by proteins containing an HD-GYP motif (Valentini and Filloux, 2016). These proteins interplay to produce and degrade c-di-GMP to maintain its proper concentration under given environmental conditions. In general, c-di-GMP stimulates biosynthesis of adhesins and inhibits various forms of motility associated with the switch from a motile planktonic lifestyle to a sedentary biofilm-associated lifestyle, whereas overproduction of EAL domain proteins induces motility (Simm et al., 2004; Römling et al., 2005, 2013; Jenal and Malone, 2006).

We used the rice pathogenic bacterium *Burkholderia glumae*, which causes panicle blight, to study functional roles of biofilm upon interaction with rice plants. Panicle blight is a rice disease that causes serious economic losses when weather conditions are favorable for the pathogen (Kim et al., 2004, 2007). We found that *B. glumae* forms cellulase-sensitive pellicles in static culture, then investigated the factors involved in pellicle formation and determined their functional roles in interactions between rice plants and *B. glumae*. One of the DGC genes present in the genome of *B. glumae* was identified to exhibit the most influence on pellicle formation. Heterologous expression of the *pleD* gene (Atu1297), whose protein is a response regulator possessing the GGDEF domain in *Agrobacterium tumefaciens*, facilitated pellicle formation in *B. glumae*. Pellicle formation was dependent upon temperature, flagella, and c-di-GMP, but independent of QS. We showed that pellicles play critical roles for colonization of *B. glumae*, thereby affecting virulence in rice plants.

## Materials and Methods

### Bacterial Strains and Growth Conditions

The bacterial strains and plasmids used in this study are listed in Supplementary Table S1. *B. glumae* and *Escherichia coli* were grown at 37°C and 250 rpm in Luria-Bertani (LB) broth containing 0.1% tryptone, 0.5% yeast extract, and 0.5% NaCl (w/v) (USB, Cleveland, OH, United States). When necessary, appropriate antibiotics were added as follows: ampicillin, 100 μg/ml; kanamycin, 50 μg/ml; tetracycline, 10 μg/ml; trimethoprim, 75 μg/ml; rifampicin, 100 μg/ml; spectinomycin, 100 μg/ml. LB agar medium contained 1.5% (w/v) agar (Becton Dickinson, Sparks, MD, United States).

### Transposon Mutagenesis and Marker Exchange

pCSR1 was mutagenized using Tn*3*-*gusA* and marker-exchanged into the wild-type strain BGR1 as described previously (Bonas et al., 1989). The sites of Tn*3-gusA* insertions were determined as described previously (Kim et al., 2004). All constructs were confirmed by Southern hybridization analysis.

### Pellicle, Swarming, and Congo Red Assays

The pellicle assay was performed at 28°C and 37°C for 3–4 days in LB broth in 24-well culture plates (Corning Inc., Corning, NY, United States) in static culture. The swarming assay was performed at 28°C on LB agar plates containing 0.7% agar as described previously (Kim et al., 2007). The Congo red binding assays were carried out at 28°C as described previously (Xu et al., 2013).

### Pellicle Degradation Enzyme Assay

Pellicles were harvested from 3- to 4-day-old cultures and washed with Dulbecco’s phosphate-buffered saline (DPBS; WELGENE, Gyeongsan, South Korea) followed by treatment with 0.86 units (U)/ml of endo-1,4-β-D-glucanase (cellulase) (Sigma-Aldrich, St. Louis, MO, United States), 0.1% proteinase K (v/v) (Sigma-Aldrich), or 0.1% RNase-free DNase I (v/v) (Qiagen, Venlo, Netherlands) as described previously (Yap et al., 2005; Liang et al., 2010). The turbidity of degraded pellicles was measured as the optical density at 600 nm (OD_600_) using an Eppendorf BioSpectrometer kinetic (Eppendorf, Hamburg, Germany) after overnight incubation at 37°C.

### Analysis of c-di-GMP

Cyclic dimeric guanosine monophosphate was extracted as described previously (Roy et al., 2013) and quantified by high-performance liquid chromatography (Dionex, Sunnyvale, CA, United States). Commercially available c-di-GMP (Sigma-Aldrich) was used as a standard.

### Expression of *pelI* in pSRKKm

To express a DGC gene with the PAS/PAC sensor (BGLU_RS21385) in the IPTG-inducible expression vector pSRKKm (Khan et al., 2008), the coding region was PCR amplified from BGR1 genomic DNA using the corresponding primers (BGLU_RS21385F, 5′-CATATGCTGACAACCGACACC-3′ and BGLU_RS21385R, 5′-AAGCTTTCACTCGCCGTACAGCTC-3′) with Phusion DNA polymerase (New England Biolabs, Beverly, MA, United States). The amplified PCR product was cloned into pGEM-T Easy (Promega, Madison, WI, United States) (Supplementary Table S1), followed by confirmation of correct sequences. The insert DNA was generated as a *Nde*I/*Spe*I fragment and cloned into its corresponding sites in pSRKKm. The plasmid pJW110 (Xu et al., 2013), which expresses *pleD* in pSRKKm, was introduced into the wild-type strain BGR1 as described previously (Figurski and Helinski, 1979).

### Plant Inoculation

The stems of rice plants (*Oryza sativa* cv. Milyang 23) were inoculated with approximately 1 × 10^8^ colony-forming units (CFU)/ml of *B. glumae* strains in a plant growth chamber (Hanbaek Scientific, Bucheon, South Korea) with a 16-h light period at 30°C and an 8-h dark period at 25°C. Rice stems were photographed every 3 days after injection. The severity of disease index was calculated using the Fiji image processing software (version 1.52o; NIH) in pixels (area of diseased surface selection in square pixel × intensity of diseased area in optical density). Colonization was evaluated daily for 9 days by enumeration of recovered cells from 3 cm above and below the inoculated sites. The virulence and colonization assays were repeated three times with three independent replicates. Analysis of variance (ANOVA)/Tukey’s correction for multiple comparison and significant difference at *P* < 0.05 were defined in disease severity and colonization assays using IBM SPSS Statistics software (version 20 × 86-x64; IBM, Armonk, NY, United States).

## Results

### Temperature- and Flagellum-Dependent, but QS-Independent, Cellulase-Sensitive Pellicle Formation

**FIGURE 1:**
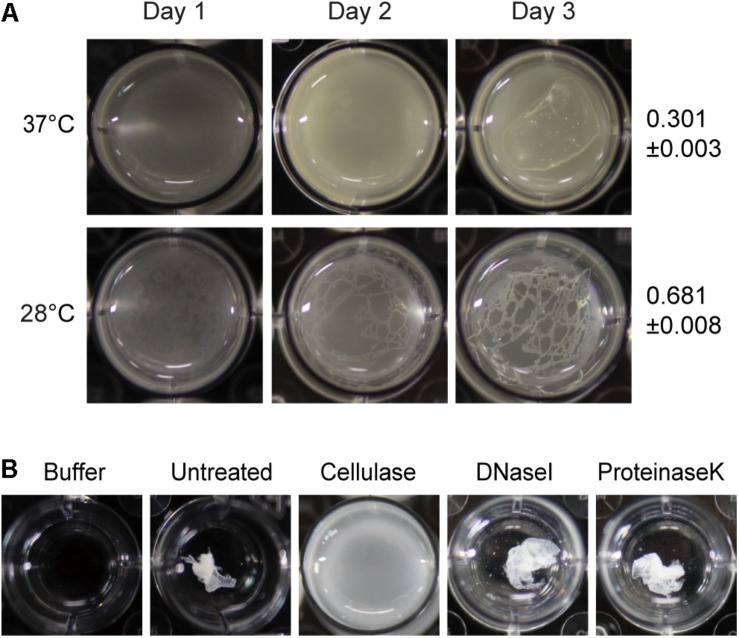
Temperature-dependent pellicle formation in *Burkholderia glumae* and enzymatic pellicle degradation. **(A)** Pellicle formation was observed for 3 days at 37°C and 28°C. A distinctive pellicle of the wild-type strain BGR1 was observed after 3 days of incubation and was more evident at 28°C than at 37°C. The turbidity of cellulase-treated pellicles from the respective temperatures was assessed on the 3rd day of incubation and is shown as the mean ± standard deviation. **(B)** The pellicle harvested from the static culture of wild-type strain BGR1 at 28°C was treated with cellulase, DNase I, or proteinase K in Dulbecco’s phosphate-buffered saline. The pellicle was degraded by the addition of cellulase, but DNase I and proteinase K did not degrade the pellicle.

To determine whether temperature affects biofilm formation, cells of *B. glumae* were incubated in 24-well plates containing LB broth supplemented with 100 mM HEPES (pH 7.0) at 37 and 28°C without shaking. Thin layers of pellicles appeared 3 days after inoculation of wild-type strain BGR1 at 37°C, whereas distinct pellicles were formed 2 days after inoculation at 28°C (Figure 1A). To determine the major component of pellicles produced by *B. glumae*, we assessed the sensitivity of pellicles to cellulase, proteinase K, and DNase I. When 4-day-old pellicles produced by wild-type strain BGR1 were treated with cellulase, the coagulated pellicles were dispersed, indicative of cellulase sensitivity, whereas no turbid dispersion was observed following treatment with 0.1% (v/v) proteinase K or 0.1% (v/v) DNase I (Figure 1B). These results indicated that a major component of pellicles is most likely composed of cellulase-sensitive materials. When 3-day-old pellicles formed at 37 and 28°C were treated with cellulase, the turbidities were 0.301 ± 0.003 (mean ± standard deviation [SD]) and 0.681 ± 0.008, respectively (Figure 1A). These results indicated that pellicle formation was more favorable at 28°C than at the optimum growth temperature of 37°C.

**FIGURE 2:**
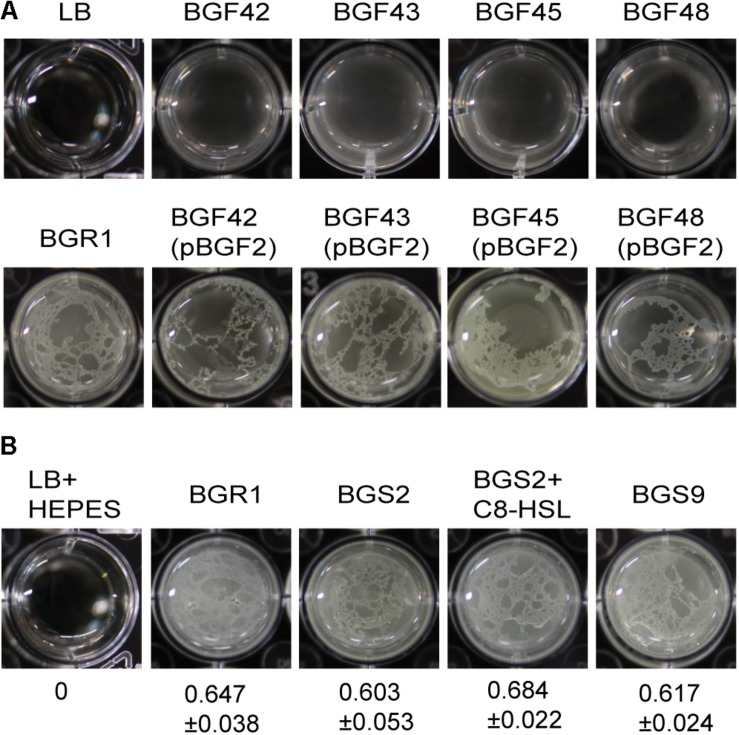
Flagella-dependent but quorum sensing-independent pellicle formation in *B. glumae*. **(A)** Pellicle formation of wild type, swimming-defective mutants, and complemented strains with pBGF2. All swimming-defective mutants, BGF42(BGR1, *flhA*:Tn*3-gusA42*), BGF43(BGR1, *cheB*:Tn*3-gusA43*), BGF45(BGR1, *fliA*:Tn*3-gusA45*), and BGF48(BGR1, *cheZ*:Tn*3-gusA48*) exhibited no pellicle formation. Genetic complementation of swimming-defective mutants with pBGF2 carrying a cluster of flagellar genes restored pellicle deficiencies in all swimming-defective mutants used. **(B)** Pellicle formation of wild-type BGR1, QS mutant BGS2(BGR1, *tofI*:Ω), BGS2 with exogenously added 1 μM C8-HSL, and *qsmR* mutant BGS9(BGR1, *qsmR*:Ω) in Luria-Bertani broth buffered with 100 mM HEPES (pH 7.0) under static culture at 28°C. Turbidity measurements at 600 nm following cellulase treatment are shown as the mean ± SD.

Because flagellum-mediated aerotactic motility is a key factor involved in pellicle formation in aerobic bacteria, we tested whether swimming motility is critical for pellicle formation in *B. glumae*. Previously characterized swimming-defective mutants BGF42(BGR1, *flhA*:Tn*3*-*gusA42*), BGF43(BGR1, *cheB*:Tn*3*-*gusA43*), BGF45(BGR1, *fliA*:Tn*3*-*gusA45*), and BGF48(BGR1, *cheZ*:Tn*3*-*gusA48*) (Supplementary Table S1) failed to form pellicles, and the genetically complemented strains recovered pellicle formation (Figure 2A). To determine whether QS is critical for pellicle formation in *B. glumae*, we performed pellicle assays with wild-type strain BGR1; two previously constructed QS-defective mutants of *B. glumae*, BGS2(BGR1, *tofI*:Ω) and BGS9(BGR1, *qsmR*:Ω) (Supplementary Table S1); and BGS2 exogenously supplemented with 1 μM *N*-octanoyl homoserine lactone (C8-HSL). The four strains did not differ in the time required for pellicle formation or the amount of pellicle produced when grown in 24-well cell culture plates containing LB broth supplemented with 100 mM HEPES (pH 7.0) at 28°C (Figure 2B). The amount of pellicle formed in 24-well culture plates was determined through OD_600_ measurement after cellulase treatment, indicated as the mean ± SD below the respective inoculum. These results indicated that pellicle formation is independent of QS in *B. glumae*.

### Identification of a Gene Cluster Comprising Putative Cellulose Biosynthetic Genes Critical for Pellicle Formation

**FIGURE 3:**
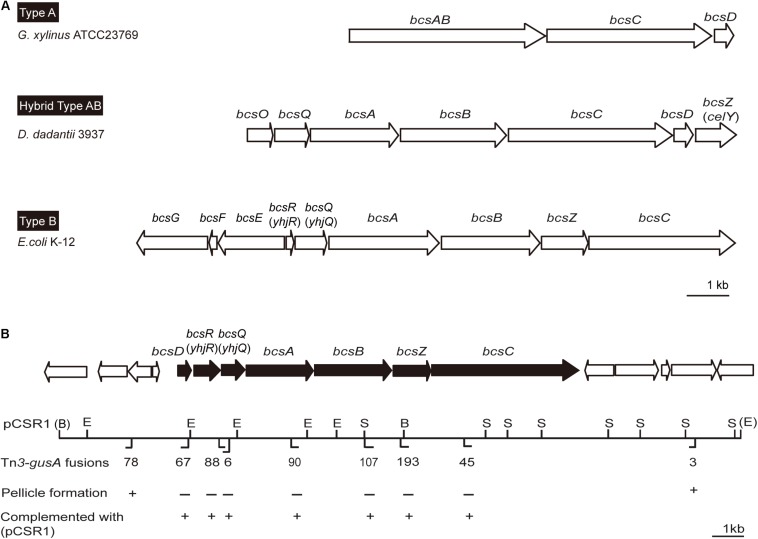
Physical maps of bacterial cellulose biosynthetic genes. **(A)** Type operons of the cellulose biosynthetic genes of pellicle-producing bacteria: type A, *G. xylinus* ATCC23769 (Saxena et al., 1994); type B, *E. coli* K-12 (Le Quéré and Ghigo, 2009); and hybrid type AB, *D. dadantii* 3937 (Jahn et al., 2011). Homologous genes are placed in vertical alignment. Open reading frames (ORFs) are drawn to scale. **(B)** Genetic map of plasmid pCSR1 including cellulose biosynthetic genes of *B. glumae* BGR1. The Tn*3-gusA* insertion and its pellicle formation (–/+) are marked below the map. B, *Bam*HI; E, *Eco*RI; and S, *Sac*I. The enzyme sites in parentheses, (B) and (E), are the enzyme sites in pLAFR3 (Supplementary Table S1). The ORFs are drawn to scale. The scale bars in **(A,B)** represent 1 kb.

**FIGURE 4:**
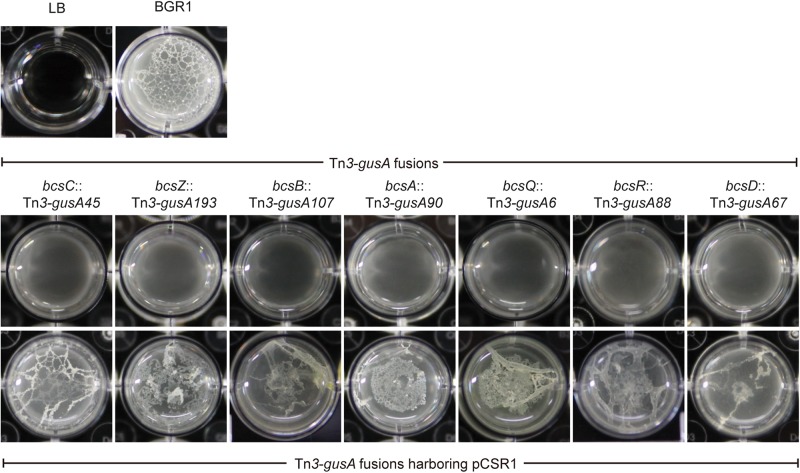
Pellicle formation of cellulose mutants and complementation strains. All cellulose mutants abolished pellicle formation and their pellicle deficiencies were recovered by complementation with plasmid pCSR1 harboring a cluster of cellulose biosynthesis genes.

Since pellicles were sensitive to cellulase, we identified genes involved in cellulose biosynthesis to determine whether cellulose biosynthesis is critical for pellicle formation. From genome information of wild-type strain BGR1, we found a gene cluster consisting of seven homologs of previously known *bcs* or regulatory genes, *bcsD* (BGLU_RS28215), *bcsR* (*yhjR*, BGLU_RS28220), *bcsQ* (*yhjQ*, BGLU_RS28225), *bcsA* (BGLU_RS28230), *bcsB* (BGLU_RS28235), *bcsZ* (*celY*, BGLU_RS28240), and *bcsC* (BGLU_RS28245) in *B. glumae* BGR1 (Figure 3A). The *bcs* homologs in BGR1 are indicated as black arrows in Figure 3B. Cosmid clone pCSR1, 22.03 kb in size, carrying the gene cluster identified from the previously constructed genomic library of *B. glumae* BGR1 was subjected to Tn*3-gusA* mutagenesis. Insertion of Tn*3-gusA* in putative cellulose biosynthetic genes was determined by direct sequencing, and each mutation was marker-exchanged into wild-type strain BGR1 to generate individual gene knockout mutants (Figure 3B). Tn*3-gusA* insertion in each of the seven genes abolished pellicle formation, and genetic complementation with pCSR1 *in trans* conferred recovery of phenotypes (Figure 4). Insertions in genes upstream and downstream of the seven *bcs* genes did not affect pellicle formation (Figure 3B). These results indicated that these seven genes are critical for cellulase-sensitive pellicle formation in *B. glumae*, despite concerns about the polar effects of Tn*3-gusA* insertion.

### Constitutive Expression of *pelI* and *pleD* Facilitated Pellicle Formation and Repressed Swarming Motility

**FIGURE 5:**
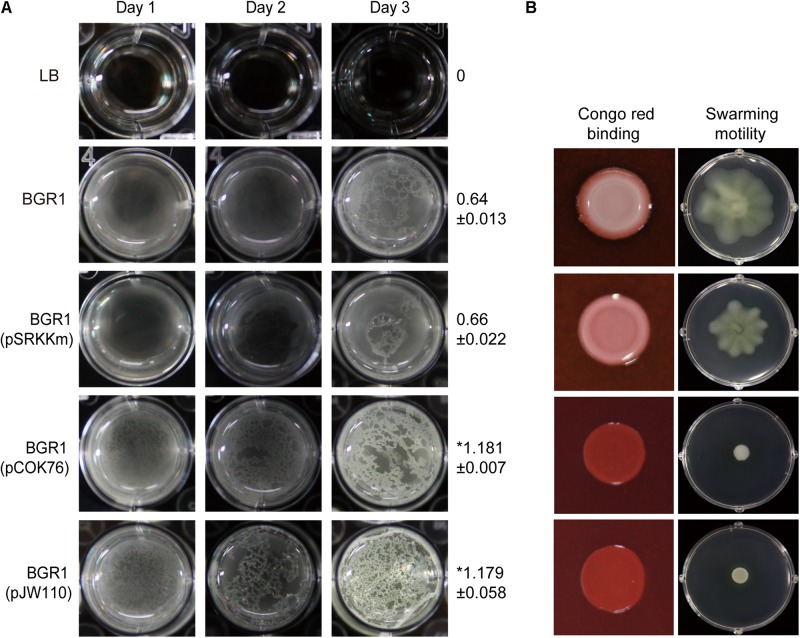
Pellicle formation, swarming motility, and Congo red binding assay. **(A)** Pellicle formation of wild-type strain BGR1, BGR1(pSRKKm, empty vector), BGR1(pCOK76; pSRKKm:*pelI*), and BGR1(pJW110; pSRKKm:*pleD*). After 3 days of incubation, pellicles were harvested, treated with cellulase, and the turbidity was measured at 600 nm. The asterisks (^∗^) represent a significant difference (*P* < 0.05) in turbidity between wild-type strain BGR1, BGR1(pCOK76), and BGR1(pJW110). **(B)** Swarming motility and Congo red binding activity of wild type BGR1, BGR1(pCOK76; pSRKKm:*pelI*), and BGR1(pJW110; pSRKKm:*pleD*).

To determine which DGC gene is the most critical for pellicle formation in *B. glumae*, we searched for genes encoding proteins possessing GGDEF, EAL, GGDEF/EAL, or HD-GYP motifs in the genome of *B. glumae* BGR1. We found 12, 7, 7, and 2 genes encoding proteins possessing GGDEF, EAL, GGDEF/EAL, and HD-GYP motifs, respectively (Supplementary Table S2). Among those, we determined that a putative DGC gene (BGLU_RS21385, designated *pelI*) possessing a PAS/GGDEF motif was the most influential for pellicle formation when constitutively expressed in the multi-copy number plasmid pCOK76 in wild-type strain BGR1 (Figure 5A). Constitutive expression of *pelI* facilitated pellicle formation as assessed by measurements of turbidity following cellulase treatment of pellicles and Congo red staining, but repressed swarming motility (Figures 5A,B). In addition, constitutive expression of a heterologous gene encoding GGDEF response regulator PleD from *A. tumefaciens* in the multi-copy number plasmid pJW110 triggered faster and denser pellicle formation compared to the wild type carrying empty vector pSRKKm. The OD_600_ of cellulase-treated pellicles harvested 3 days after inoculation was measured, and the turbidity values of saturated pellicles of BGR1 (wild type), BGR1(pSRKKm), BGR1(pCOK76), and BGR1(pJW110) in cellulase DPBS (v/v) were 0.64 ± 0.013, 0.66 ± 0.022, 1.181 ± 0.007, and 1.179 ± 0.058, respectively (Figure 5A). Constitutive expression of *pleD* repressed swarming motility in *B. glumae* (Figure 5B). These results indicated that DGC genes are critically involved in pellicle biosynthesis in *B. glumae*.

**FIGURE 6:**
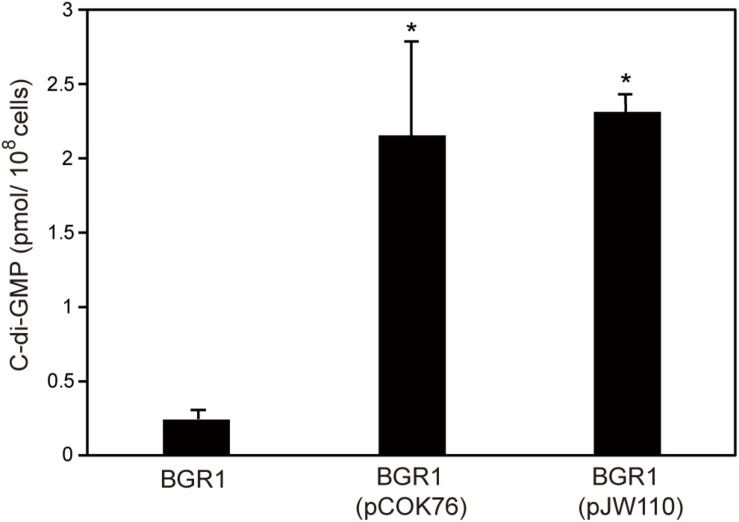
Quantification of c-di-GMP in wild-type strain BGR1, BGR1(pCOK76; pSRKKm:*pelI*), and BGR1(pJW110; pSRKKm:*pleD*) using LC/MS analysis. BGR1(pCOK76; pSRKKm:*pelI*) and BGR1(pJW110; pSRKKm:*pleD*) strains exhibited increased production of c-di-GMP. The asterisks (^∗^) represent a significant difference (*P* < 0.05) in c-di-GMP among wild-type strain BGR1, BGR1(pCOK76), and BGR1(pJW110).

To confirm that constitutive expression of *pelI* and *pleD* in *B. glumae* affected levels of c-di-GMP, we measured the levels of c-di-GMP present in wild-type strain BGR1, BGR1 carrying *pelI* in pCOK76, and BGR1 carrying *pleD* in pJW110. c-di-GMP levels were significantly higher in BGR1 carrying pCOK76 or pJW110 than in wild-type strain BGR1 (Figure 6). These results were in good agreement with the fact that c-di-GMP is a key signal molecule for biofilm biosynthesis in bacteria.

### Pellicle-Defective Mutants Were Less Virulent

**FIGURE 7:**
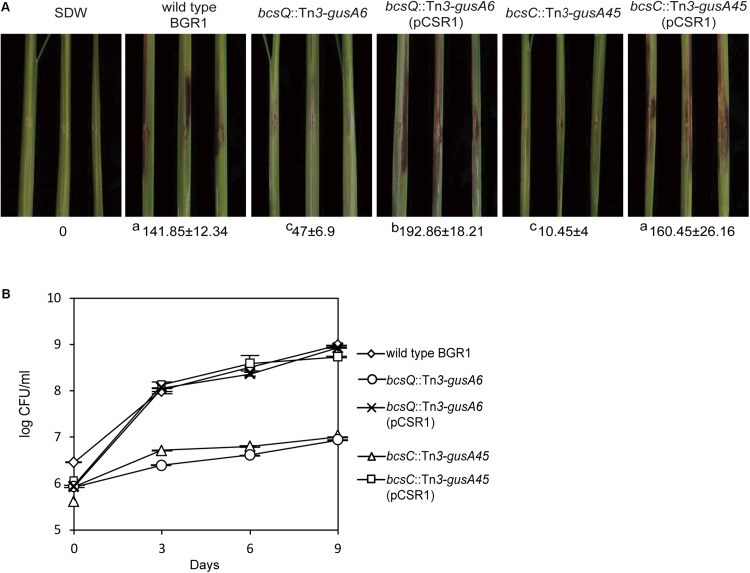
Virulence of wild type, non-pellicle producing cellulose mutants, and complementation strains in rice sheath. **(A)** The pellicle-defective mutants with Tn*3-gusA* insertion in putative cellulose biosynthetic genes, *bcsQ* and *bcsC*, exhibited no serious symptoms in rice sheaths compared to the wild type, with a score value of 141.85 ± 12.34. The virulence of non-pellicle producing cellulose mutants was recovered by complementation with pCSR1. The diseased areas in wild type, non-pellicle producing cellulose mutants, and complemented strains were scored in pixels with SDs. The superscripts (a, b, and c) before the mean values indicate significant differences (*P* < 0.05) based on ANOVA/Tukey’s correction for multiple comparisons. **(B)** Changes in population of wild type, non-pellicle producing cellulose mutants, and complementation strains in the inoculated sheath. Non-pellicle producing cellulose mutants exhibited significant differences compared to the wild type. Population density in non-pellicle producing cellulose mutants was recovered by complementation with *bcs* operon-carrying pCSR1. The error bars represent three independent inoculation experiments. Two representative data are shown in this figure; the others are shown in Supplementary Figure S1.

To determine the functional role of cellulose-sensitive pellicles of *B. glumae* in rice plants, we inoculated pellicle-forming and non-pellicle-forming cells into stems of rice plants. The pellicle defective mutants with Tn*3-gusA* insertions in genes involved in cellulose biosynthesis caused no serious damage to rice sheaths when compared to the wild type (Figure 7A and Supplementary Figure S1A). Colonization of pellicle-defective mutants was significantly less effective than that of wild-type strain BGR1 for the 9-day period after inoculation (Figure 7B and Supplementary Figure S1B). Complementation of pellicle-defective mutants with pCSR1 carrying the seven putative cellulose biosynthetic genes fully recovered the colonization ability and virulence (Figures 7A,B, and Supplementary Figures S1A,B). These results indicated that defects in forming cellulase-sensitive pellicles reduce the colonization ability of *B. glumae* in rice plants, thereby affecting virulence.

## Discussion

The ability to sense the available oxygen is critical for aerobes to switch their lifestyle and become sessile at the air–liquid interface and thus to form pellicles in static culture conditions (Hölscher et al., 2015; Kovács and Dragoš, 2019). While oxygen and various environmental factors are known to influence bacterial pellicle formation, little information is available as to whether temperature affects bacterial pellicle formation. Most pellicle assays are routinely performed at the optimum growth temperature of the bacterium in question. However, this does not necessarily mean that the conventional methods used for biofilm assays always apply in all cases. The physiological conditions and microenvironments of hosts when bacteria associate with their hosts should be critical for their initial interactions and colonization. In this regard, our results revealed such cases where plant pathogenic bacteria interact with their hosts. The fact that the optimal temperature for pellicle formation was different from that for growth supports the importance of temperature during interactions between plant pathogenic bacteria and their hosts. Our data showed that pellicle formation is critical for the initial colonization of *B. glumae* in rice tissues, as assessed by monitoring population changes with time after inoculation. However, it was not determined whether pellicles were actually formed in rice stems by direct observation. As to mechanisms involved in the role of pellicles for colonization in *B. glumae*, we assume that cellulase-sensitive pellicles might be helpful for initial contact and adhesion of *B. glumae* cells in rice tissues.

Flagellum-mediated bacterial motility is necessary for aerobes to migrate toward oxygen-rich environments in static culture conditions, but is not a requirement for pellicle development (Houry et al., 2010; Yamamoto et al., 2012; Hölscher et al., 2015). The fact that aerotactic motility accelerates pellicle formation in static culture was consistent with our observation that swimming-defective mutants fail to develop pellicles in *B. glumae*. Since bacterial QS has been known to play an important role in biofilm formation in gram-negative bacteria (De Kievit, 2009), we initially hypothesized that pellicle formation might be regulated by QS. However, QS mutants formed pellicles in buffered LB static culture conditions. The observation that pellicle formation was independent of QS was unexpected because flagellar biosynthesis is positively controlled by QS (Kim et al., 2007; Jang et al., 2014). This contradiction can be explained in two ways. First, actual flagellar biosynthesis might occur differently between flagellum-defective mutants due to null mutations in flagellar biosynthetic genes and QS-negative strains in which expression of genes involved in flagellar biosynthesis is not activated. Second, considering that bacterial pellicle formation is triggered by unfavorable environments to make adjustments for survival in gram-negative bacteria, unfavorable laboratory culture conditions such as lack of aeration and non-optimal growth temperatures might have a more crucial impact on triggering pellicle proliferation than QS in *B. glumae*.

Genetic variations in *bcs* genes were grouped into type A and B (Jahn et al., 2011). A representative bacterium of type A is *Gluconacetobacter xylinus*, which is used for industrial cellulose production (Jahn et al., 2011). A single operon consisting of four genes, *bcsABCD*, is responsible for cellulose biosynthesis in *G. xylinus* (Jahn et al., 2011). BcsA and B are responsible for polymerization of glycan chains whereas BcsC and BcsD are involved in glucan extrusion and crystallization during cellulose assembly (Saxena et al., 1994). In type B, *bcsZ* is present between *bcsB* and *bcsC*, and there is an additional operon, *bcsEFG*, in *E. coli*, *Pectobacterium atrosepticum*, and *Salmonella enterica* (Le Quéré and Ghigo, 2009; Jahn et al., 2011). The genetic organization of the *bcs* gene cluster in *B. glumae* has unique features that do not belong to type A or B. The *bcs* gene cluster of *B. glumae* has the *bcsD* gene typical of type A, but also has *bcsZ* between *bcsB* and *bcsC*, and *bcsR*, unique to type B, which shows intermediate gene organization between type A and B. Such a hybrid form of *bcs* gene organization has been found in the plant pathogenic bacterium *Dickeya dadantii* (Prigent-Combaret et al., 2012). It would not be surprising to find more variations in the genetic organization of *bcs* gene clusters present in bacteria originating from diverse environmental niches.

Cyclic dimeric guanosine monophosphate plays an important role as a second messenger in bacteria. However, we often encounter difficulties in finding the most influential genes involved in c-di-GMP biosynthesis and its degradation due to the presence of multiple copies of DGCs and PDEs (Römling and Amikam, 2006; Hengge, 2009; Morgan et al., 2014). Therefore, heterologous but well-characterized DGCs are often used to study c-di-GMP-mediated signaling in target bacteria. We initially confirmed that pellicle formation is controlled by c-di-GMP by adapting *pleD* from *A. tumefaciens*, and then identified the most critical gene, *pelI*, among 21 putative DGC genes in *B. glumae*. These results do not add new information to biofilm research, but it is worth finding the most influential DGC gene among many gene families in *B. glumae*.

Enhanced adhesion ability of rhizosphere colonizing *Pseudomonas putida* and *Pseudomonas fluorescens* via elevated levels of c-di-GMP to plant roots occurred due to facilitated biofilm formation by elevated c-di-GMP levels (Newell et al., 2009; Matilla et al., 2011). Likewise, it is likely that pellicle formation would be helpful for initial contact and adhesion of *B. glumae* to rice tissues, which eventually would affect the colonization ability and virulence. Nonetheless, it remains to be answered whether pellicles or biofilms produced by *B. glumae* truly exist around the infection sites of rice tissues. In addition to our previous reports of QS-mediated virulence mechanisms of *B. glumae* (Kim et al., 2004, 2007), pellicle was found to be an important virulence factor independent of QS. This finding may provide a new drug target to control rice panicle blight caused by *B. glumae*.

## Data Availability Statement

All datasets generated for this study are included in the article/Supplementary Material.

## Author Contributions

G-YK, JK, and IH conceived and designed the study. G-YK, OC, EG, and YK carried out the experiments. G-YK, OC, EG, YK, JK, and IH analyzed and interpreted the data. G-YK and IH wrote the manuscript.

## Conflict of Interest

The authors declare that the research was conducted in the absence of any commercial or financial relationships that could be construed as a potential conflict of interest.
