# Supplementary material for: Quorum Sensing-Independent Cellulase-Sensitive Pellicle Formation Is Critical for Colonization of Burkholderia glumae in Rice Plants
Source: Front Microbiol. 2020 Jan 17;10:3090. doi: 10.3389/fmicb.2019.03090 (PMC6978641; doi:10.3389/fmicb.2019.03090)
Supplement: Supplementary file 1 [file Data_Sheet_1.PDF]

Quorum sensing-independent cellulase-sensitive pellicle formation is critical for colonization of *Burkholderia glumae* in rice plants

***Gi-Young Kwak*[1]*, Okhee Choi*[2]*, Eunhye Goo*[1]*, Yongsung Kang*[1]*, Jinwoo Kim*[2*] *and Ingyu Hwang*[1,3*]**

[1]*Department of Agricultural Biotechnology, Seoul National University, Seoul 08826, Republic of Korea*

[2]*Division of Applied Life Science and Institute of Agriculture and Life Sciences, Gyeongsang National University, Jinju 52828, Republic of Korea*

[3]*Research Institute of Agriculture and Life Sciences, Seoul National University, Seoul 08826, Republic of Korea*

***Correspondence:**

Jinwoo Kim, Ingyu Hwang

jinwoo@gnu.ac.kr; ingyu@snu.ac.kr

Table of Contents

Figure S1. Virulence and colonization assays of all mutated and complemented strains for seven putative cellulose biosynthesis regulatory genes in pSCR1..……………….…………Page 2–3

Table S1. Strains and plasmids used in this study……………………………………..…Page 4–5

Table S2. Identified GGDEF/EAL domain encoding genes in *B. glumae* ……….……..Page 6–7

Supplemental references for Table S1……….……………………………………………..Page 7–8

A

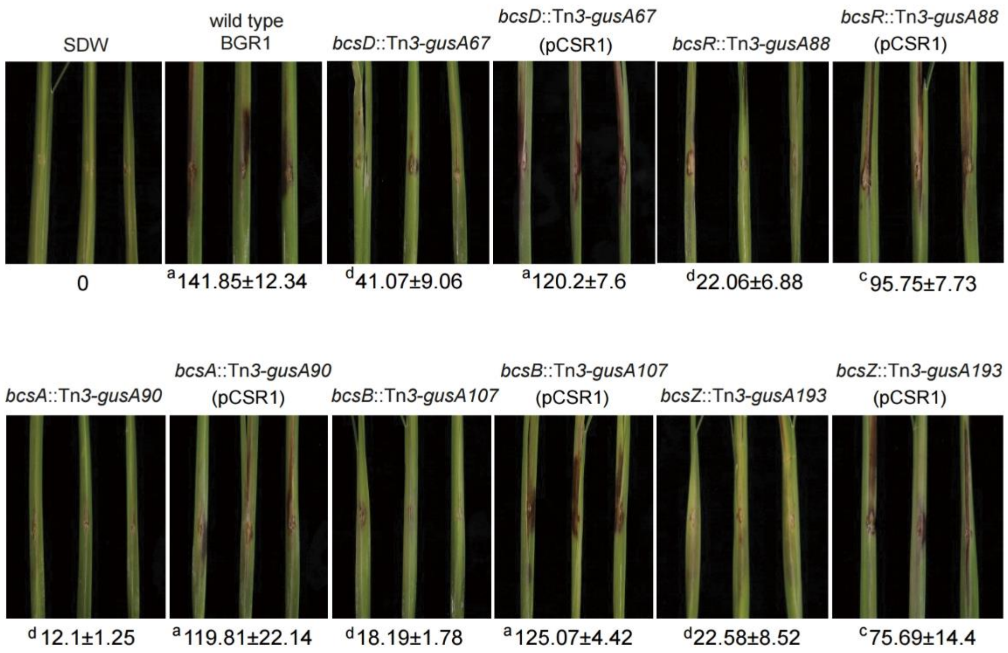
SDW
wild type BGR1
bcsD::Tn3-gusA67
bcsD::Tn3-gusA67 (pCSR1)
bcsR::Tn3-gusA88
bcsR::Tn3-gusA88 (pCSR1)
0
a141.85±12.34
d41.07±9.06
a120.2±7.6
d22.06±6.88
c95.75±7.73
bcsA::Tn3-gusA90
bcsA::Tn3-gusA90 (pCSR1)
bcsB::Tn3-gusA107
bcsB::Tn3-gusA107 (pCSR1)
bcsZ::Tn3-gusA193
bcsZ::Tn3-gusA193 (pCSR1)
d12.1±1.25
a119.81±22.14
d18.19±1.78
a125.07±4.42
d22.58±8.52
c75.69±14.4

B

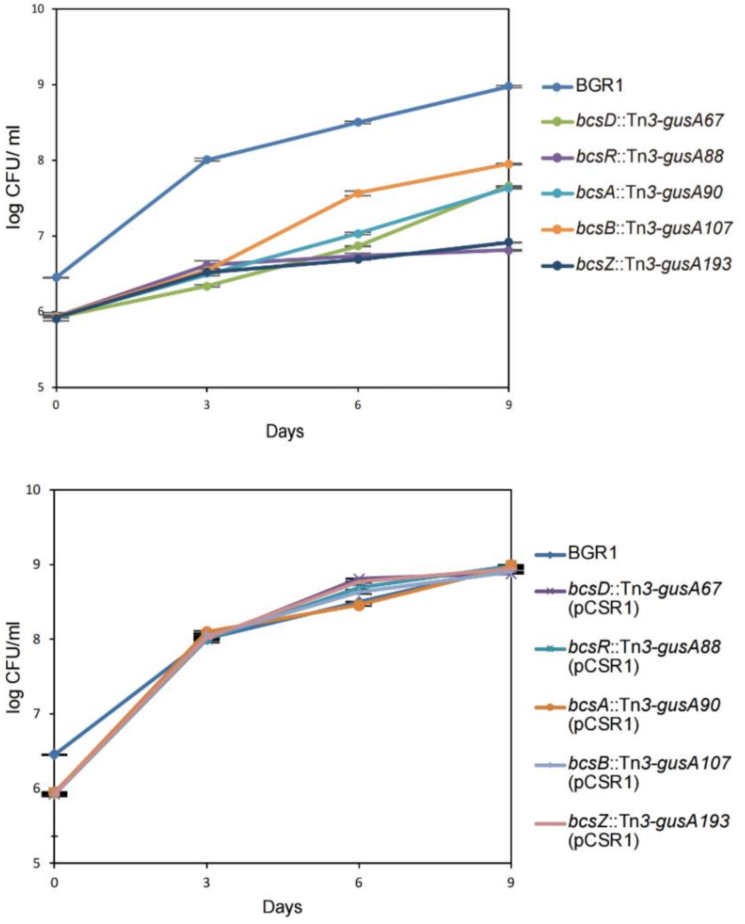
log CFU/ ml
Days
BGR1
bcsD::Tn3-gusA67
bcsR::Tn3-gusA88
bcsA::Tn3-gusA90
bcsB::Tn3-gusA107
bcsZ::Tn3-gusA193
log CFU/ml
Days
BGR1
bcsD::Tn3-gusA67 (pCSR1)
bcsR::Tn3-gusA88 (pCSR1)
bcsA::Tn3-gusA90 (pCSR1)
bcsB::Tn3-gusA107 (pCSR1)
bcsZ::Tn3-gusA193 (pCSR1)

Figure S1. Virulence assay of non-pellicle forming cellulose mutant strains. **(A)** The pellicle defective mutants with Tn*3*-*gusA* insertion in putative cellulose biosynthetic genes, *bcsD*, *R*, *A*, *B*, and *Z*, exhibited no serious symptoms in rice sheaths compared to wild-type strain BGR1. The complemented strains exhibited full recovery of virulence. The superscripts (a, b, c, and d) on the mean values represent a significant difference ($p < 0.05$) based on ANOVA/Tukey's correction for multiple comparisons in virulence scores in pixels among treatments. Values are presented as the mean ± standard deviation. **(B)** Changes in population of wild type, non-pellicle producing cellulose mutants, and complementation strains in rice sheaths. The colonization ability of the cellulose mutants with restored pellicle production was recovered to the level of wild-type strain BGR1.

Table S1. Strains and plasmids used in this study.

Bacterial strain or plasmid	Genotype	Source or reference
Strains		
Burkholderia glumae		
BGR1	Wild-type, Rif^R	(Jeong et al., 2003)
BGS2	BGR1, *tofI*::Ω	(Kim et al., 2004)
BGS9	BGR1, *qsmR*::Ω	(Kim et al., 2007)
BGA90	BGR1, *bcsA*::Tn*3-gusA90*	This study
BGB107	BGR1, *bcsB*::Tn*3-gusA107*	This study
BGC45	BGR1, *bcsC*::Tn*3-gusA45*	This study
BGD67	BGR1, *bcsD*::Tn*3-gusA67*	This study
BGZ193	BGR1, *bcsZ*::Tn*3-gusA193*	This study
BGQ6	BGR1, *bcsQ*::Tn*3-gusA6*	This study
BGR88	BGR1, *bcsR*::Tn*3-gusA88*	This study
BGF42	BGR1, *flhA*::Tn*3-gusA42*	(Kim et al., 2007)
BGF43	BGR1, *cheB*::Tn*3-gusA43*	(Kim et al., 2007)
BGF45	BGR1, *fliA*::Tn*3-gusA45*	(Kim et al., 2007)
BGF48	BGR1, *cheZ*::Tn*3-gusA48*	(Kim et al., 2007)
Escherichia coli		
DH5α	F^- Φ80d*lacZ*Δ*M15*Δ(*lacZYA-argF*) *U169* endA1 *recA1 hsd1 hsdR17*($r_k^- m_k^+$) *deoR thi-1supE44*λ$^-$ *gyrA96 relA1*	Gibco BRL
S17-1	Tra$^+$, *recA*, Sp^R	(Simon et al., 1983)
C2110	*polA*, Nal^R	(Stachel et al., 1985)

Plasmids		
pSRKKm	pBBR1MCS-2-derived broad-host-range expression vector containing lac promoter and lacI^q and lacZα$^+$, Km^R	(Khan et al., 2008)
pBluescript II SK(+)	pUC derivative, Amp^R	Stratagene
pLysS	Encodes T7 lysozyme gene, Cm^R	Novagen
pSShe	Cm^R	(Stachel et al., 1985)
pHoKmGus	Promoterless *β*-glucuronidase gene, Km^R, Amp^R *tnpA*	(Bonas et al., 1989)
pRK2013	Tra$^+$, ColE1 replicon, Km^R	(Figurski and Helinski, 1979)
pLAFR3	Tra$^-$, Mob$^+$, RK2 replicon, Tet^R	(Staskawicz et al., 1987)
pJW110	pSRKKm carrying P_{lac}-*pleD*, Km^R	(Xu et al., 2013)
pCOK76	Plasmid vector carrying *lacI^q*, P*lac*, *lacZα*, and *pelI*, Km^R	This study
pCSR1	Plasmid harboring *bcsA*, *B*, *C*, *D*, *Z*, and *yhjQ*	This study
pBGF2	23 kb DNA fragment harboring *fliA*, *flhA*, *CheZ*, and *CheB* from BGR1 cloned into pLAFR3	(Kim et al., 2007)

Table S2. Identified GGDEF/EAL domain encoding genes in *B. glumae.*

Locus ID*	Domain	Annotation
BGLU_RS01045	REC/GGEEF	Diguanylate cyclase 6
BGLU_RS05070	PAC/GGDEF/EAL	Diguanylate cyclase/phosphodiesterase (GGDEF & EAL domains) with PAS/PAC sensor(s)
BGLU_RS05175	EAL	Hypothetical protein BDAG_01993
BGLU_RS06570	GGDEF/EAL	Diguanylate cyclase/phosphodiesterase with PAS/PAC sensor (frame shifted)
BGLU_RS09190	GGDEF/EAL	Diguanylate cyclase 3
BGLU_RS12055	EAL/HDOD	Diguanylate phosphodiesterase2/*Burkholderia cenocepacia* pBCA055
BGLU_RS13960	GGDEF/EAL	Hypothetical protein BMASAVP1 A1055
BGLU_RS14170	GGDEF/EAL	Diguanylate cyclase/phosphodiesterase 2
BGLU_RS17000	EAL	Cyclic diguanylate phosphodiesterase
BGLU_RS17085	GGDEF/EAL	Diguanylate cyclase/phosphodiesterase
BGLU_RS17210	GGDEF	Porin, Gram-negative type
BGLU_RS18985	GGDEF	Hypothetical protein BB4664
BGLU_RS21375	GGDEF/EAL	Glycogen debranching enzyme Glgx 4
BGLU_RS21385	PAS/GGDEF	Diguanylate cyclase with PAS/PAC sensor (*pelI*)
BGLU_RS22710	GGDEF/EAL	Thioester reductase
BGLU_RS24205	GGDEF	Allophanate hydrolase subunit
BGLU_RS25010	GGDEF/EAL	Diguanylate cyclase/phosphodiesterase 4

BGLU_RS25390	GGDEF	Glycogen debranching enzyme Glgx2
BGLU_RS27365	EAL	Diguanylate phosphodiesterase 4
BGLU_RS28165	EAL	Diguanylate phosphodiesterase 3
BGLU_RS28585	GGDEF/EAL	Diguanylate cyclase/phosphodiesterase 3
BGLU_RS29240	HD-GYP	Metal-dependent phosphohydrolase
BGLU_RS17205	HD	Metal-dependent phosphohydrolase

*Locus IDs were obtained from GenBank.

Supplemental References

Bonas, U., Stall, R. E., and Staskawicz, B. (1989). Genetic and structural characterization of the avirulence gene *AvrBs3* from *Xanthomonas campestris* pv. *vesicatoria*. *Mol. Gen. Genet.* 218, 127–136.

Figurski, D. H., and Helinski, D. R. (1979). Replication of an origin-containing derivative of plasmid RK2 dependent on a plasmid function provided in *trans*. *Proc. Natl. Acad. Sci. U. S. A.* 76, 1648–1652. doi: 10.1073/pnas.76.4.1648

Jeong, Y., Kim, J., Kim, S., Kang, Y., Nagamatsu, T., Hwang I. (2003). Toxoflavin produced by *Burkholderia glumae* causing rice grain rot is responsible for inducing bacterial wilt in many field crops. *Plant Dis*. 87, 890–895. doi: 10.1094/PDIS.2003.87.8.890

Khan, S. R., Gaines, J., Roop, R. M. 2nd and Farrand, S. K. (2008). Broad-host-range expression vectors with tightly regulated promoters and their use to examine the influence of TraR and TraM expression on Ti plasmid quorum sensing. *Appl. Environ. Microbiol.* 74, 5053–5062. doi: 10.1128/AEM.01098-08

Kim, J., Kang, Y., Choi, O., Jeong, Y., Jeong, J. E., Lim, J. Y., et al. (2007). Regulation of polar flagellum genes is mediated by quorum sensing and FlhDC in *Burkholderia glumae*. *Mol. Microbiol.* 64, 165–179. doi: 10.1111/j.1365-2958.2007.05646.x

Kim, J., Kim, J. G, Kang, Y., Jang, J. Y., Jog, G. J., Lim, J. Y., et al. (2004). Quorum sensing and the LysR-type transcriptional activator ToxR regulate toxoflavin biosynthesis and transport in *Burkholderia glumae*. *Mol. Microbiol.* 54, 921–934. doi: 10.1111/j.1365-2958.2004.04338.x

Simon, R., Priefer, U., and Pühler, A. (1983). A broad host range mobilization system for *in vivo* genetic engineering: Transposon mutagenesis in gram-negative bacteria. *Nat. Biotechnol*. 1, 784–791. https://doi.org/10.1038/nbt1183-784

Stachel, S. E., An, G., Flores, C., and Nester, E. W. (1985). A Tn*3lacZ* transposon for the random generation of beta-galactosidase gene fusions: application to the analysis of gene expression in *Agrobacterium*. *EMBO J.* 4, 891–898.

Staskawicz, B., Dahlbeck, D., Keen, N., and Napoli, C. (1987). Molecular characterization of cloned avirulence genes from race 0 and race 1 of *Pseudomonas syringae* pv. *glycinea*. *J. Bacteriol*. 169, 5789–5794. doi: 10.1128/jb.169.12.5789-5794.1987

Xu, J., Kim, J., Koestler, B. J., Choi, J. H., Waters, C. M., and Fuqua, C. (2013). Genetic analysis of *Agrobacterium tumefaciens* unipolar polysaccharide production reveals complex integrated control of the motile-to-sessile switch. *Mol. Microbiol.* 89, 929–948. doi: 10.1111/mmi.12321
